# Plasma vitamin D and parathormone are associated with obesity and atherogenic dyslipidemia: a cross-sectional study

**DOI:** 10.1186/1475-2840-11-149

**Published:** 2012-12-11

**Authors:** Alba Guasch, Mònica Bulló, Antoni Rabassa, Anna Bonada, Daniel Del Castillo, Fàtima Sabench, Jordi Salas-Salvadó

**Affiliations:** 1 Human Nutrition Unit, Faculty of Medicine and Health Sciences, IISPV, Universitat Rovira i Virgili, Reus, Spain; 2 Human Nutrition and Dietetics, Hospital Universitari Sant Joan de Reus, IISPV, Universitat Rovira i Virgili, Reus, Spain; 3 CIBERobn Physiopathology of Obesity and Nutrition, Institute of Health Carlos III, Madrid, Spain; 4 Surgery Service, Hospital Universitari Sant Joan de Reus, IISPV, Universitat Rovira i Virgili, Reus, Spain; 5 Human Nutrition Unit. Faculty of Medicine and Health Sciences, Universitat Rovira i Virgili, C/ Sant Llorenç 21, Reus, 43201, Spain

**Keywords:** Obesity, Metabolic syndrome, Vitamin D, Parathormone

## Abstract

**Background:**

Low concentrations of plasma vitamin D (25(OH)D) have been associated with the development of metabolic syndrome (MetS), obesity, diabetes and cardiovascular disease. The objective of this study was to quantify the associations between 25(OH)D and parathormone (PTH) plasma levels and obesity, the presence of MetS, diabetes or atherogenic dyslipidemia (AD) in a large sample of individuals with different degrees of adiposity.

**Methods:**

Retrospective study of all patients who had attended the obesity clinics in a Spanish hospital between 2009 and 2011, and whose concentrations of PTH, 25(OH)D, calcium and alkaline phosphatase had been determined (n=316, 75.9% women). Individuals were categorized by degree of adiposity, presence of MetS, and other comorbidities.

**Results:**

PTH increased but 25(OH)D and calcium decreased with increasing adiposity. The prevalence of 25(OH)D deficiency or insufficiency increased with obesity (<10% when BMI<45kg/m^2^, and 26% when >50). The prevalence of hyperparathyroidism increased from 12% in non-obese to 47.5% in morbidly obese individuals with BMI>50 kg/m^2^. Low plasma 25(OH)D and high PTH concentrations were associated with an increased risk of MetS and AD. These associations disappeared, except in the case of AD for 25(OH)D when adjusting for BMI. Regression analysis revealed that BMI and age or seasonality were independent predictors of PTH and 25(OH)D levels, respectively.

**Conclusions:**

BMI was the variable most strongly associated with plasma 25(OH)D and PTH concentrations in our study. Low 25(OH)D and high PTH concentrations were not independently associated with an increased risk of MetS, or diabetes. Our data support a possible contribution of plasma 25(OH)D to the pathogenesis of hypertriglyceridemia and AD through inflammation.

## Background

Obesity has become a serious health problem in most developed countries. The World Health Organization estimated that in 2008 the global prevalence of overweight and obesity was around 1.5 billion and 500 million adults, respectively. Obesity is also well known to be associated with an increase in the prevalence of type 2 diabetes, cardiovascular diseases, certain types of cancer and total mortality [1].

Vitamin D deficiency is also an important worldwide public health problem [2]. Although the most-studied and best-known function of vitamin D, together with parathyroid hormone (PTH), is related to bone metabolism [3], many studies show evidence of the relationship between obesity and low levels of 25(OH)D (the best indicator of clinical levels of vitamin D) [4,5]. Factors known to influence 25(OH)D concentrations include race, vitamin D intake, sun exposure, adiposity, age and physical activity [3]. In obese people, low levels of 25(OH)D can be attributed mainly to: a) the lower bioavailability of the vitamin, due to its sequestration by adipose tissue [6]; b) the dilution of ingested or cutaneously synthesized vitamin D in the enlarged fat mass [7]; c) low sun exposure, due to mobility limitations or the low sun exposure of large areas of the body [8]; or d) a low intake of calcium and vitamin D. The frequently observed increases in PTH serum concentrations in obese individuals [9] could be explained by a compensatory mechanism in response to low circulating levels of 25(OH)D.

More recently, vitamin D deficiency has been related to the pathogenesis of such comorbidities as insulin resistance [10], type 2 diabetes [11], hypertension [12], dyslipidemia [13], and cardiovascular diseases [14]. A controversial association has been reported between low levels of plasma 25(OH)D and/or elevated PTH and the presence of metabolic syndrome (MetS) and its individual components,. Some cross-sectional studies note that decreased levels of 25(OH)D are associated to a higher risk of MetS after adjusting for various potential confounding factors [15,16]. A recent systematic review and meta-analysis showed that the prevalence of MetS was reduced by approximately 50% if individuals had high 25(OH)D concentrations [17]. Other studies showed that high concentrations of PTH, but not 25(OH)D, were associated with the presence of the syndrome [18,19]. However, no association has been reported between PTH or 25(OH)D and MetS in overweight or obese individuals [20].

In this context, the aim of our study was to evaluate the associations between 25(OH)D or PTH concentrations and the risk of obesity, and MetS and its individual components in a large sample of individuals with a wide range of adiposity.

## Methods

### Study population

The medical records of all patients attending the obesity outpatient clinics of the Hospital Sant Joan de Reus between September 2009 and December 2011 were retrospectively reviewed. Caucasian individuals who had had at least one blood test that simultaneously measured plasma concentrations of PTH, 25(OH)D, calcium, phosphate and alkaline phosphatase were included in the present study.

Exclusion criteria were bariatric surgery before the blood test, any severe chronic illness, drug or alcohol addiction, endocrine disease (except type 2 diabetes mellitus or treated hyperthyroidism), cancer in the previous 5 years, active concomitant inflammatory disease, advanced liver disease, chronic renal failure and continued drug treatment with derivatives of vitamin D and calcium. Subjects were also excluded when primary hyperparathyroidism was suspected, when plasma concentrations of PTH were high (> 6.85 pmol/L) and hypercalcemia (> 2.5 mmol/L) and/or hypophosphatemia (<0.8 mmol/L) were present simultaneously. The study was conducted in agreement with the 1990 Declaration of Helsinki and subsequent amendments. The Sant Joan Hospital Ethical Committee from Reus (Spain) approved the study protocol.

### Design

A cross-sectional study was conducted on a population of individuals with a wide range of adiposity. From the medical records of the individuals selected we collected the following data: age, gender, current smoking (absent/present), presence of comorbidities associated with obesity (type 2 diabetes, hypertension, dyslipidemia), medication use (fibrates, statins and other lipid lowering agents, oral antidiabetic agents, insulin and antihypertensive drugs) and seasonality of blood sampling (winter: December-February, spring: March-May, summer: June-August, autumn: September-November). Weight and height were measured with individuals wearing light clothing and without shoes using calibrated scales and a wall-mounted stadiometer (scale capacity of 220 kg and a precision of 50 g, and a measuring range between 60–200 cm with an accuracy of 1 mm). BMI was calculated by dividing the weight (in kg) by the square of the height (in meters). Blood pressure was measured with a mercury sphygmomanometer adapted to the subject’s BMI, and after the subject had been seated for at least five minutes.

Individuals were classified into different categories of adiposity using the BMI-based index of the Spanish Society for the Study of Obesity (SEEDO) [21]. The MetS was defined according to the updated NCEP-ATP III criteria [22], which require three or more of the following conditions to be met: abdominal obesity (waist circumference >102 cm in men and >88 cm in women), hypertriglyceridemia (triglyceride level 150 mg/dL or 1.7 mmol/L or taking triglyceride-lowering medication), low HDL cholesterol (<40 mg/dL or 1.0 mmol/L in men and <50 mg/dL or 1.3 mmol/L in women), elevated fasting blood glucose (100 mg/dL or 5.6 mmol/L or taking antidiabetic medication), and elevated blood pressure (systolic 130 mm Hg, diastolic 85 mm Hg, or taking antihypertensive medication). Participants who were being treated with antidiabetic, antihypertensive or triglyceride-lowering medication were considered to be diabetic, hypertensive, or hypertriglyceridemic, respectively. Atherogenic dyslipidemia was defined when high plasma triglycerides and low HDL-concentrations were concomitantly present. We considered that those patients for whom we had no waist circumference measurement complied with the criterion of abdominal obesity when their BMI was higher than 35 kg/m^2^.

### Biochemical determinations

Blood samples were obtained after an overnight fast. Plasma concentrations of glucose, calcium, phosphate, alkaline phosphatase, total cholesterol, HDL cholesterol, LDL cholesterol, triglycerides, creatinine, serum albumin, erythrocyte sedimentation rate (ESR) and leukocyte count were determined following routine biochemical laboratory protocols. The corrected calcium level based on the serum albumin concentrations was calculated as: Corrected calcium (mmol/L) = measured total Ca (mmol/L) + 0.02 (40 - serum albumin [g/L]), where 40 represents the average albumin level in g/L. The ultrasensitive C Reactive Protein (uCRP) was determined by immunoturbidimetry. Serum 25(OH)D was determined by electrochemiluminiscence immunoassay using a Cobas autoanalyzer (Roche Diagnostics, West Sussex, UK; intra- and inter-assay CVs <8% and <10%, respectively). Intact PTH were determined by chemiluminescent enzyme-labeled immunometric assay using an IMMULITE 2000 Systems analyzer (Siemens, Gwynedd, UK; intra- and inter-assay CVs <5% and <8%, respectively).

The normal, insufficient and deficient vitamin D statuses were defined as serum concentrations of 25(OH)D >75.00 nmol/L, between 25.00 and 75.00 nmol/L, and <25.00 nmol/L, respectively [23]. Secondary hyperparathyroidism was diagnosed in individuals with serum concentrations of PTH > 6.85 pmol/L.

### Statistical analysis

Data are given as means (95% confidence interval, 95% CI) for continuous variables or percentages for categorical variables, unless otherwise stated. Differences between groups were tested using one way analysis of variance (ANOVA) for continuous data and the chi-square test (χ^2^) for categorical data. The studied population was divided into six BMI ranges (<30, 30–35, 35–40, 40–45, 45–50 and >50 Kg/m^2^). To evaluate predictors of 25(OH)D and PTH, a regression analysis was performed with age, gender, BMI, plasma HDL and LDL cholesterol, triglycerides, glucose, the season when the blood sample was obtained [categorized as summer (March to August) or winter (September to February)], and presence of hypertension as covariates (Model 1). In a separate analysis (Model 2), uCRP and leucocytes were added to Model 1 as covariates.

Multiple logistic regressions with predefined explanatory variables were used to assess odds for MetS, components of the MetS and atherogenic dyslipidemia. Four separate multiple logistic regression models were fitted. In the first model, serum PTH, 25(OH)D and calcium were entered into a multiple logistic regression analysis with MetS (yes/no), presence of MetS components (yes/no) and atherogenic dyslipidemia (yes/no) as dependent variables. In the second model, age, gender, season of blood sampling and current smoking were added to model 1 as confounding variables. In the third model (model 3), BMI was also included. In the fourth model, uCRP and leucocytes were included. A statistical significance level of 5% was chosen. The analyses were implemented using the SPSS 19.0 statistical package.

## Results

The general characteristics of the studied population are shown in Table 1. A total of 316 patients met the inclusion criteria and were included in the analyses. Of these 75.9% (n = 240) were women. The prevalence of MetS was 62.0%, with no significant differences between the sexes. High blood pressure was the most common feature of MetS observed in both sexes. Blood pressure and plasma concentrations of glucose, triglycerides, albumin and creatinine were significantly higher, and plasma HDL and LDL cholesterol, and ESR were lower in men than in women. Women showed lower plasma concentrations of 25(OH)D and calcium than males.

**Table 1:** Characteristics of the study population

**Variables**	**Total (n=316)**	**Men (n=76)**	**Women (n=240)**	P*
Age (years)	46.85 (45.44, 48.26)	49.28 (46.31, 52.24)	46.08 (44.49, 47.68)	0.056
Weight (Kg)	105.29 (102.30, 108.28)	111.06 (103.77, 118.36)	103.46 (100.28, 106.65)	0.032
BMI (Kg/m^2^)	40.02 (38.94, 41.09)	37.67 (35.29, 40.05)	40.76 (39.56, 41.96)	0.016
**Presence of metabolic syndrome**	62.0%	61.8%	62.1%	1.000
Abdominal obesity	69.9%	50.0%	75.8%	<0.001
Elevated blood pressure	83.4%	91.4%	80.4%	0.039
Elevated fasting glucose	55.6%	61.3%	53.8%	0.287
Reduced HDL cholesterol	61.8%	54.2%	64.1%	0.165
Elevated fasting triglycerides	30.0%	45.1%	25.4%	0.003
Current smoking	20.6%	21.1%	20.4%	0.620
Seasonality (spring-summer)	39.2%	40.8%	38.8%	0.788
**Drugs**				
Fibrates	2.3%	4.3%	1.7%	0.195
Oral antidiabetic	18.0%	27.6%	15.0%	0.016
Insulin	2.8%	5.3%	2.1%	0.226
Antihypertensive	34.2%	48.7%	29.6%	0.003
Systolic blood pressure (mmHg)	139.99 (137.35, 142.63)	146.39 (140.83, 151.95)	137.61 (134.68, 140.54)	0.003
Diastolic blood pressure (mmHg)	84.01 (82.50, 85.52)	87.68 (84.88, 90.47)	82.65 (80.89, 84.41)	0.003
Glucose (mmol/L)	6.05 (5.83, 6.27)	6.57 (5.99, 7.15)	5.88 (5.66, 6.11)	0.009
HDL cholesterol (mmol/L)	1.22 (1.17, 1.26)	1.06 (0.97, 1.15)	1.27 (1.21, 1.32)	<0.001
LDL cholesterol (mmol/L)	3.22 (3.11, 3.32)	2.91 (2.68, 3.14)	3.31 (3.19, 3.42)	0.002
Triglycerides (mmol/L)	1.48 (1.37, 1.58)	1.74 (1.45, 2.02)	1.39 (1.29, 1.49)	0.005
ALAT (μKat/L)	0.36 (0.34, 0.38)	0.41 (0.37, 0.45)	0.35 (0.33, 0.37)	0.014
GT (μKat/L)	0.44 (0.40, 0.49)	0.59 (0.45, 0.73)	0.41 (0.36, 0.45)	0.001
Leucocyte count (x10E9/L)	7.69 (7.43, 7.95)	7.43 (6.75, 8.10)	7.76 (7.47, 8.04)	0.324
Albumin (g/L)	40.25 (39.70, 40.81)	42.04 (40.66, 43.41)	39.80 (39.21, 40.39)	0.001
CRP ultrasensitive (mg/dL)	9.41 (8.30, 10.52)	8.53 (5.37, 11.69)	9.62 (8.46, 10.79)	0.444
ESR (mm/h)	22.67 (20.61, 24.74)	13.10 (10.04, 16.17)	25.03 (22.68, 27.38)	<0.001
Calcium (mmol/L)	2.34 (2.33, 2.35)	2.36 (2.34, 2.38)	2.33 (2.32, 2.34)	0.020
Phosphate (mmol/L)	1.16 (1.13, 1.18)	1.13 (1.08, 1.18)	1.16 (1.14, 1.19)	0.294
Alkaline phosphatase (uKat/L)	1.23 (1.17, 1.29)	1.21 (1.06, 1.36)	1.24 (1.17, 1.31)	0.656
Creatinine (mol/L)	63.33 (61.55, 65.12)	80.00 (75.81, 84.20)	59.19 (57.63, 60.74)	<0.001
eGFR (ml/min/1.73m^2^)	103.08 (101.16, 105.20)	98.86 (93.89, 103.83)	104.25 (102.06, 106.44)	0.035
PTH (pmol/L)	5.83 (5.53, 6.14)	5.42 (4.84, 5.99)	5.97 (5.61, 6.32)	0.129
25(OH)D (nmol/L)	56.28 (51.58, 60.98)	65.01 (53.18, 76.83)	53.55 (48.60, 58.49)	0.041

Data are means (95% CI) for continuous variables and % for categorical variables.

*P value comparing males and females: One-way analysis of variance (ANOVA) for continuous variables, and chi-square test (χ^2^) for categorical variables.

Abbreviations: *BMI*: Body Mass Index; *HDL*: High Density Lipoprotein; *LDL*: Low Density Lipoprotein; *ALAT*: Alanine transaminase; *GT*: Glutamyl transaminase; *CRP*: C Reactive Protein; *ESR*: Erythrocyte Sedimentation Rate; *eGFR*: Estimated Glomerular Filtration Rate; *PTH* Parathormone; *25(OH)D*; 25 Hydroxy-cholecalcipherol.

Table 2 describes the characteristics of the patients in the various BMI ranges. A higher proportion of women were observed among those with a BMI higher than 35 kg/m^2^. The prevalence of MetS and its features (abdominal obesity, elevated blood pressure, hyperglycemia, and low HDL-c) increased when the BMI range was increased. Although there were no significant differences in blood pressure, LDL cholesterol and plasma triglycerides across BMI ranges, plasma glucose, ultrasensitive uCRP, ESR and leukocyte counts increased and HDL cholesterol and albumin decreased when the BMI range increased.

**Table 2:** **Characteristics of patients according to body mass index (kg/m**^**2**^**) ranges**

	**BMI (Kg/m**^**2**^**)**						
	**<30 (n=50)**	**30-35 (n=45)**	**35-40 (n=45)**	**40-45 (n=81)**	**45-50 (n=53)**	**>50 (n=42)**	P*
Age (years)	45.2 (40.2, 50.2)	53.0 (49.3, 56.7) ^#^	44.8 (41.4, 48.3)	45.99 (43.5, 48.5)	46.1 (42.9, 49.2)	47.3 (44.3, 50.4)	0.022
Women	60.8%	60.0%	80.0%	82.7%	84.9%	83.3%	0.002
Weight (Kg)	66.6 (63.3, 69.9)	87.2 (84.4, 90.1) ^##^	99.5 (95.9, 103.1) ^##^	112.2 (109.4, 114.9) ^##^	122.2 (118.5, 125.8) ^##^	143.1 (136.2, 145.00) ^##^	<0.001
BMI (Kg/m^2^)	24.9 (23.9, 26.00)	32.4 (31.9, 32.8) ^##^	37.3 (36.8, 37.7) ^##^	42.7 (42.4, 43.0) ^##^	47.2 (46.8, 47.6) ^##^	55.2 (53.5, 56.8) ^##^	<0.001
**Presence of MetS**	19.6%	35.6%	57.8%	79.0%	79.2%	92.9%	<0.001
Abdominal obesity	0.0%	4.4%	100.0%	100.0%	100.0%	100.0%	<0.001
Elevated blood pressure	59.2%	83.3%	78.8%	93.5%	92.7%	93.9%	<0.001
Elevated fasting glucose	31.4%	55.8%	45.5%	60.0%	63.5%	78.6%	<0.001
Reduced HDL cholesterol	14.0%	50.0%	60.5%	76.6%	80.4%	83.3%	<0.001
Elevated fasting triglycerides	25.5%	39.0%	27.9%	25.0%	35.5%	33.3%	0.556
Systolic blood pressure (mmHg)	136.4 (128.5, 144.3)	141.4 (136.00, 146.8)	133.8 (127.4, 140.2)	142.4 (137.5, 147.4)	141.5 (136.1, 147.0)	144.2 (136.2, 152.2)	0.244
Diastolic blood pressure (mmHg)	80.1 (76.6, 83.7)	85.6 (81.8, 89.3)	82.4 (77.9, 87.00)	85.3 (82.5, 88.2)	84.4 (80.2, 88.7)	87.0 (82.8, 91.2)	0.087
Glucose (mmol/L)	5.34 (4.88, 5.79)	6.05 (5.59, 6.51)	5.80 (5.35, 6.26)	6.08 (5.63, 6.53)	6.13 (5.59, 6.68)	7.09 (6.21, 7.97) ^##^	0.002
HDL cholesterol (mmol/L)	1.60 (1.47, 1.73)	1.21 (1.11, 1.32) ^##^	1.24 (1.11, 1.36) ^##^	1.12 (1.05, 1.20) ^##^	1.07 (0.97, 1.18) ^##^	1.10 (1.02, 1.17) ^##^	<0.001
LDL cholesterol (mmol/L)	2.87 (2.58, 3.16)	3.27 (2.95, 3.59)	3.45 (3.18, 3.72)	3.19 (3.01, 3.36)	3.32 (3.05, 3.59)	3.23 (2.93, 3.53)	0.070
Triglycerides (mmol/L)	1.26 (0.99, 1.54)	1.56 (1.25, 1.87)	1.54 (1.13, 1.95)	1.43 (1.28, 1.58)	1.61 (1.40, 1.82)	1.52 (1.26, 1.77)	0.464
ALAT (μKat/L)	0.35 (0.29, 0.41)	0.32 (0.30, 0.35)	0.35 (0.32, 0.38)	0.36 (0.33, 0.39)	0.38 (0.31, 0.45)	0.40 (0.34, 0.45)	0.430
GT (μKat/L)	0.27 (0.21, 0.32)	0.45 (0.27, 0.63)	0.47 (0.35, 0.59)	0.48 (0.38, 0.59)	0.44 (0.37, 0.50)	0.48 (0.39, 0.57)	0.131
Leucocyte count (x10E9/L)	6.08 (5.47, 6.70)	6.17 (5.65, 6.70)	7.93 (7.12, 8.74) ^#^	7.99 (7.53, 8.44) ^##^	8.26 (7.66, 8.86) ^##^	8.63 (8.01, 9.25) ^##^	<0.001
Albumin (g/L)	47.0 (45.5, 48.5)	41.9 (40.4, 43.3) ^##^	40.6 (39.4, 41.8) ^##^	38.8 (38.00, 39.6) ^##^	38.4 (37.3, 39.5) ^##^	38.8 (37.5, 40.1) ^##^	<0.001
CRP ultrasensitive (mg/dL)	2.67 (1.90, 3.45)	4.17 (2.71, 5.63)	7.75 (5.81, 9.68)	9.35 (7.27, 11.44) ^#^	11.16 (8.77, 13.54) ^#^	16.80 (13.07, 20.53) ^##^	<0.001
ESR (mm/h)	14.50 (10.15, 18.85)	18.74 (14.56, 22.92)	18.38 (13.70, 23.05)	21.03 (17.93, 24.12)	26.95 (21.94, 31.96) ^#^	34.84 (27.16, 42.52) ^##^	<0.001
25(OH)D (nmol/L)	95.8 (76.0, 115.5)	59.1 (48.5, 69.7) ^##^	48.2 (42.3, 54.0) ^##^	49.8 (42.6, 57.1) ^##^	46.6 (40.0, 53.2) ^##^	37.6 (30.3, 44.8) ^##^	<0.001
PTH (pmol/L)	4.27 (3.61, 4.93)	5.11 (4.50, 5.72)	5.84 (5.07, 6.61)	6.05 (5.50, 6.60) ^#^	6.39 (5.68, 7.10) ^#^	7.41 (6.31, 8.52) ^##^	<0.001
Calcium (mmol/L)	2.38 (2.34, 2.41)	2.32 (2.29, 2.35)	2.32 (2.30, 2.35)	2.33 (2.31, 2.35)	2.33 (2.31, 2.36)	2.34 (2.31, 2.36)	0.046
Phosphate (mmol/L)	1.20 (1.12, 1.27)	1.11 (1.03, 1.19)	1.19 (1.13, 1.26)	1.14 (1.10, 1.18)	1.17 (1.11, 1.22)	1.16 (1.10, 1.21)	0.374
Alkaline phosphatase (uKat/L)	1.14 (1.04, 1.25)	1.13 (1.02, 1.24)	1.30 (1.15, 1.46)	1.23 (1.10, 1.35)	1.21 (1.03, 1.38)	1.42 (1.19, 1.64)	0.132
eGFR (ml/min/1.73m^2^)	105.1 (99.3, 110.8)	99.1 (91.6, 106.6)	101.9 (97.1, 106.6)	105.0 (101.7, 108.4)	103.2 (98.0, 108.5)	102.4 (96.7, 108.1)	0.627

Data are means (CI) for continuous variables and % for categorical variables.

*P value by one-way analysis of variance (ANOVA) for continuous variables, and chi-square test (χ^2^) for categorical variables.

# Statistical significance compared to the first BMI group (BMI<30 Kg/m^2^): ^#^P <0.05 and ^##^P <0.001 by ANOVA, Bonferroni posthoc test.

Abbreviations: *BMI*: Body Mass Index; *MetS*: Metabolic Syndrome; *HDL*: High Density Lipoprotein; *LDL*: Low Density Lipoprotein; *ALAT*: Alanine transaminase; *GT*: Glutamyl transaminase; *uCRP*: ultrasensitive C Reactive Protein; *ESR*: Erythrocyte Sedimentation Rate; *PTH* Parathormone; *25(OH)D*; 25.Hydroxy-cholecalcipherol; *eGFR*: Estimated Glomerular Filtration Rate.

Significant mean differences in plasma calcium (P<0.046), 25(OH)D (P<0.001) and PTH (P<0.001) concentrations were observed across the individuals categorized by BMI. Individuals with lower BMI had higher plasma concentrations of 25(OH)D and calcium than individuals with higher BMI (Table 2). The concentrations of PTH increased as BMI ranges increased. No mean differences were found in plasma phosphate and alkaline phosphatase concentrations between BMI groups.

Table 3 shows the vitamin D status and the prevalence of secondary hyperparathyroidism for the BMI categories. The prevalence of vitamin D deficiency and insufficiency increased with obesity (by <10% in individuals with BMI <45 kg/m^2^, and 26% in those >50 kg/m^2^). When vitamin D deficiency and insufficiency were merged, only 38% of individuals with a BMI lower than 30 kg/m^2^ had vitamin D insufficiency or deficiency, compared to 88-95% of those with a BMI higher than 35 kg/m^2^. The prevalence of hyperparathyroidism increased from 12% in non-obese subjects to 47.5% in those with a BMI >50 kg/m^2^.

**Table 3:** **Vitamin D status and presence of hyperparathyroidism according to body mass index (kg/m**^**2**^**) ranges**

	**BMI (kg/m**^**2**^**)**						
	**<30 (n=50)**	**30-35 (n=45)**	**35-40 (n=45)**	**40-45 (n=81)**	**45-50 (n=53)**	**>50 (n=42)**	P*
**Vitamin D status**							
Deficient	8.0%	9.1%	9.1%	9.1%	12.0%	26.2%	<0.001
Insufficient	30.0%	63.6%	81.8%	80.5%	76.0%	69.0%	
Optimal	56.0%	27.3%	9.1%	10.4%	12.0%	4.8%	
**Hyperparathyroidism**	12.0%	15.6%	37.5%	29.9%	36.0%	47.5%	0.001

* P value from the Chi-square test (χ^2^).

Abbreviations: *BMI*: Body Mass Index.

Vitamin D status by 25(OH)D concentration: deficient (< 25 nmol/L), insufficient (25–75 nmol/L) and optimal (>75 nmol/L).

Hyperparathyroidism: parathyroid hormone concentration > 6.85 pmol/L.

In a multivariate analysis (Table 4), BMI, age and HDL-cholesterol were independent predictors of serum PTH levels, and together they explain 21% of the variance in PTH concentrations. For plasma 25(OH)D concentrations, BMI and blood sample seasonality were the independent predictors, explaining 17% of their variability. When uCRP and leukocyte counts were added to the model, BMI and uCRP levels explained 12% of the variability of 25(OH)D concentrations.

**Table 4:** Predictors of PTH and 25(OH)D by multiple regression analysis

**Model 1**	**β**	**95% CI**	P*	**R**^**2**^	**Model 2**	**β**	**95% CI**	P*	**R**^**2**^
**PTH (pmol/L) (n=229)**					**PTH (pmol/L) (n=180)**				
BMI (kg/m^2^)	0.331	0.045, 0.119	<0.001		BMI (kg/m^2^)	0.291	0.032 – 0.129	0.001	
Age (years)	0.263	0.025, 0.079	<0.001		Age (years)	0.187	0.007, 0.070	0.018	
Gender	0.050	−0.471, 1.097	0.433		Gender	0.062	−0.609, 1.438	0.425	
c-HDL (mmol/L)	−0.162	−1.959, -0.129	0.026		c-HDL (mmol/L)	−0.171	−2.403, -0.029	0.045	
c-LDL (mmol/L)	0.077	−0.140, 0.584	0.228	0.211	c-LDL (mmol/L)	0.067	−0.235, 0.625	0.373	
TG (mmol/L)	−0.093	−0.851, 0.141	0.159		TG (mmol/L)	−0.134	−1.114, 0.103	0.103	0.146
Glucose (mmol/L)	−0.092	−0.301, 0.058	0.183		Glucose (mmol/L)	−0.077	−0.308, 0.110	0.350	
Seasonality	−0.138	−1.415, -0.102	0.024		Seasonality	−0.101	−1.316, 0.221	0.162	
Hypertension	0.031	−0.735, 1.168	0.654		Hypertension	0.044	−0.803, 1.420	0.584	
					CRP (mg/dL)	−0.034	−0.060, 0.039	0.672	
					Leucocyte count (x10E9/L)	0.027	−0.153, 0.218	0.731	
**25(OH)D (nmol/L) (n=234)**					**25(OH)D (nmol/L) (n=186)**				
BMI (kg/m^2^)	−0.324	−2.103, -0.801	<0.001		BMI (kg/m^2^)	−0.238	−1.357, -0.212	0.008	
Seasonality	−0.151	−25.459, -2.834	0.014		CRP (mg/dL)	−0.195	−1.289, -0.133	0.016	
Age (years)	0.032	−0.341, 0.560	0.633		Age (years)	0.013	−0.334, 0.395	0.869	
Gender	−0.112	−25.299, 1.781	0.088		Gender	−0.039	−15.284, 9.172	0.622	
c-HDL (mmol/L)	0.079	−7.362, 25.292	0.280	0.171	c-HDL (mmol/L)	0.010	−13.590, 15.323	0.906	
c-LDL (mmol/L)	−0.007	−6.617, 5.911	0.912		c-LDL (mmol/L)	−0.114	−8.973, 1.195	0.133	0.120
TG (mmol/L)	−0.081	−13.394, 3.268	0.232		TG (mmol/L)	−0.088	−10.637, 3.121	0.282	
Glucose (mmol/L)	0.017	−2.624, 3.347	0.812		Glucose (mmol/L)	0.076	−1.251, 3.478	0.354	
Hypertension	0.014	−15.052, 18.421	0.843		Seasonality	−0.130	−17.153, 0.731	0.072	
					Hypertension	−0.030	−15.748, 10.713	0.708	
					Leucocyte count (x10E9/L)	0.109	−0.652 – 3.745	0.167	

* P value by multiple regression analysis.

Abbreviations: *PTH* Parathormone; *BMI*: Body Mass Index; *25(OH)D*: 25.Hydroxy-cholecalcipherol *CRP*: C Reactive Protein.

Model 1. Potential predictors are age, gender, BMI, c-HDL, c-LDL, TG, glucose, season and presence of hypertension.

Model 2. Potential predictors are age, gender, BMI, c-HDL, c-LDL, TG, glucose, season, presence of hypertension, CRP and leucocyte count.

In a multiple logistic regression (Table 5), PTH levels were associated with significantly higher odds of MetS in model 1, but not after adjustment for other confounders (models 2, 3 and 4). PTH was also positively associated with elevated blood pressure and reduced HDL cholesterol components of the MetS (models 1 and 2). However, these associations disappeared when adjusted for BMI. PTH was associated with higher odds of atherogenic dyslipidemia in both model 1 and model 2, but not after adjustment for BMI (model 3). High 25(OH)D levels were significantly associated with lower odds of MetS, and reduced HDL cholesterol and diabetes/hyperglycemia components of the MetS in models 1 or 2, but not after the final adjustment for BMI (models 3 and 4). High 25(OH)D levels were significantly associated with lower odds of elevated triglyceride components of the MetS even after adjustment for several confounding factors.

**Table 5:** Odds for prevalent metabolic syndrome, metabolic syndrome components and atherogenic dyslipidemia according to levels of PTH and 25(OH)D

**Dependent variable**	**Model I**		**Model 2**		**Model 3**		**Model 4**	
**Explanatory variables**	**OR (95% CI)**	P*	**OR (95% CI)**	P*	**OR (95% CI)**	P*	**OR (95% CI)**	P*
**Metabolic syndrome**								
PTH	1.13 (1.01, 1.26)	0.027	1.10 (0.98, 1.24)	0.090	0.93 (0.81, 1.07)	0.309	0.99 (0.83, 1.17)	0.880
25(OH)	0.99 (0.98, 1.00)	0.004	0.99 (0.98, 1.00)	0.004	1.00 (0.99, 1.01)	0.825	1.00 (0.99, 1.02)	0.690
Age			1.02 (1.00, 1.05)	0.027	1.04 (1.01, 1.07)	0.005	1.04 (1.01, 1.08)	0.011
BMI					1.20 (1.14, 1.26)	<0.001	1.21 (1.13, 1.30)	<0.001
**Elevated blood pressure**								
PTH	1.40 (1.17, 1.68)	<0.001	1.26 (1.03, 1.54)	0.027	1.10 (0.88, 1.39)	0.409	1.15 (0.88, 1.51)	0.297
25(OH)D	1.00 (0.99, 1.00)	0.219	0.99 (0.98, 1.00)	0.141	1.00 (0.99, 1.01)	0.677	1.00 (0.98, 1.01)	0.825
Age			1.07 (1.04, 1.10)	<0.001	1.06 (1.03, 1.10)	<0.001	1.03 (0.99, 1.07)	0.117
BMI					1.13 (1.06, 1.20)	<0.001	1.14 (1.05, 1.23)	0.002
**Reduced HDL cholesterol**								
PTH	1.13 (1.01, 1.27)	0.031	1.16 (1.03, 1.31)	0.015	1.05 (0.92, 1.19)	0.446	1.07 (0.93, 1.24)	0.352
25(OH)D	0.99 (0.98, 0.99)	0.001	0.99 (0.98, 0.99)	0.001	0.99 (0.99, 1.00)	0.180	1.00 (0.98, 1.01)	0.618
BMI					1.11 (1.07, 1.16)	<0.001	1.09 (1.04, 1.14)	<0.001
**Elevated fasting triglycerides**								
PTH	0.93 (0.84, 1.04)	0.208	0.92 (0.82, 1.03)	0.136	0.90 (0.80, 1.01)	0.080	0.86 (0.74, 1.00)	0.051
25(OH)D	0.99 (0.98, 1.00)	0.037	0.99 (0.98, 1.00)	0.024	0.99 (0.98, 1.00)	0.060	0.98 (0.97, 1.00)	0.026
Calcium Tertile 1	0.50 (0.26, 0.97)	0.045	0.52 (0.26, 1.05)	0.068	0.53 (0.27, 1.08)	0.079	0.71 (0.30, 1.65)	0.423
Tertile 2	0.51 (0.27, 0.97)	0.040	0.51 (0.26, 0.99)	0.047	0.50 (0.25, 0.97)	0.041	1.00 (0.45, 2.22)	0.998
**Diabetes**								
PTH	1.10 (0.99, 1.22)	0.072	1.02 (0.91, 1.15)	0.733	0.93 (0.82, 1.06)	0.270	0.94 (0.82, 1.01)	0.420
25(OH)D	0.99 (0.99, 1.00)	0.078	0.99 (0.99, 1.00)	0.023	1.00 (0.99, 1.00)	0.449	1.00 (0.99, 1.01)	0.787
Calcium Tertile 1	0.35 (0.19, 0.66)	0.001	0.41 (0.20, 0.84)	0.014	0.45 (0.22, 0.93)	0.032	0.46 (0.19, 1.09)	0.079
Tertile 2	0.44 (0.24, 0.80)	0.008	0.48 (0.24, 0.94)	0.034	0.43 (0.21, 0.88)	0.021	0.48 (0.20, 1.12)	0.088
Age			1.07 (1.05, 1.10)	<0.001	1.09 (1.06, 1.12)	<0.001	1.10 (1.06, 1.14)	<0.001
BMI					1.09 (1.05, 1.13)	<0.001	1.07 (1.02, 1.12)	0.005
**Atherogenic dyslipidemia**								
PTH	1.22 (1.07, 1.40)	0.004	1.18 (1.02, 1.36)	0.022	1.07 (0.92, 1.25)	0.345	1.02 (0.87, 1.20)	0.798
25(OH)D	0.99 (0.98, 0.99)	<0.001	0.99 (0.98, 0.99)	<0.001	0.99 (0.98, 1.00)	0.032	0.99 (0.98, 1.01)	0.407
Calcium Tertile 1	0.36 (0.16, 0.80)	0.012	0.41 (0.18, 0.92)	0.032	0.41 (0.17, 0.96)	0.040	0.50 (0.19, 1.29)	0.153
Tertile 2	0.41 (0.19, 0.87)	0.021	0.43 (0.19, 0.93)	0.033	0.34 (0.14, 0.80)	0.014	0.46 (0.18, 1.17)	0.102
BMI					1.09 (1.05, 1.14)	<0.001	1.11 (1.05, 1.17)	<0.001

*P value by multiple logistic regression analysis.

PTH: Parathormone; 25(OH)D: 25.Hydroxy-cholecalcipherol; BMI: Body mass index.

Model 1. Explanatory variables in the multiple logistic regression model were PTH, 25(OH)D and tertiles of calcium.

Model 2. Explanatory variables as in model 1 plus age, gender, season and current smoking.

Model 3. Explanatory variables as in model 2 plus BMI.

Model 4. Explanatory variables as in model 3 plus uCRP and leukocyte count.

Higher 25(OH)D levels were associated with significantly lower odds of having atherogenic dyslipidemia (models 1, 2 and 3). This association also disappeared when the model was additionally adjusted for uCRP concentrations and leukocyte count.

## Discussion

The most important finding of the present study is that both 25(OH)D and PTH were strongly associated with adiposity but not with MetS or most of its components. Several other studies have suggested that low 25(OH)D status is associated with the development of the MetS and its individual components. However, our data do not support an independent contribution of 25(OH)D or PTH to the pathogenesis of the MetS in a population with a wide range of adiposity. Our data are also consistent with previous reports on the high prevalence of alterations in calcium metabolism [5,24] in obese subjects.

Traditionally, vitamin D has been the key regulator of serum calcium metabolism either directly or indirectly through PTH. However, vitamin D receptors are found in a wide variety of tissue, including gut, adipose tissue, cardiac and skeletal muscles, and β-cells [25]. Therefore, it is not surprising that numerous studies have investigated the potential key role of vitamin D in the pathogenesis of MetS and its individual components. As stated in the introduction section, some but not all epidemiologic studies conducted in general populations have demonstrated a relationship between low plasma 25(OH)D concentrations and the presence of MetS or its individual components [15-17]. Although these epidemiological observations are supported by mechanistic studies, experimental data are limited, especially in obese populations.

In this regard, a recent cross-sectional study conducted on 380 individuals, more than 80% of whom were overweight or obese, showed a strong association between plasma 25(OH)D concentrations and BMI [26]. Individuals with 25(OH)D deficiency had higher odds of MetS than those who had normal 25(OH)D status, probably because the authors failed to account for the confounding effect of BMI on the association between MetS and 25(OH)D. In this study, no associations between plasma 25(OH)D concentrations and the individual components of MetS were shown [26].

Similar data were obtained by Botella-Carretero *et al*. [27] in a Spanish study conducted in a reduced sample of severely obese subjects. In this study, 25(OH)D deficiency was more prevalent in patients with the MetS than in those without. In contrast, Hjelmesaeth *et al*. [19], and Roislien et al. 2011 [28], failed to find any association between 25(OH)D and MetS, but reported a positive relationship between PTH plasma levels and MetS in individuals with morbid obesity. Neither was an association found between PTH or 25(OH)D and MetS in overweight or obese individuals from New Zealand [20] or Spanish morbidly obese patients [29].

There is also some controversy about the effect of low 25(OH)D status on the pathogenesis of obesity-related metabolic comorbidities and individual components of the MetS [30]. As far as glucose metabolism is concerned, Forouhi *et al*. [31] used data from the Medical Research Council Ely Prospective Study to show that 25(OH)D concentrations at baseline were inversely related with a 10-fold risk of hyperglycemia and insulin resistance. Recent studies also suggest that increased levels of PTH are independently associated with insulin resistance in individuals with abdominal obesity suggesting a direct link between PTH and MetS [32].

Similar results have been found in many observational studies [33,34]. In contrast, the results of intervention studies are inconclusive, often because they involve the joint administration of vitamin D_3_ (cholecalciferol) and Ca. This makes it difficult to interpret the results, because it is not clear whether the response observed is due to vitamin D and/or Ca. In some randomized clinical trials (RCTs) in which cholecalciferol was used as a single treatment, insulin response to glucose improved [35,36], but in others it did not. Similar findings were observed in relation to blood pressure and hypertension. Despite strong mechanistic evidence, the results of epidemiologic studies evaluating the association between 25(OH)D status and blood pressure are contradictory and inconclusive. The odds of having high blood pressure were lower in Australian individuals with higher 25(OH)D concentrations than in those with lower concentrations [37]. In contrast, 25(OH)D did not predict future risk of hypertension or increases in blood pressure in the Tromso Study [38]. Only one RCT using cholecalciferol has been conducted to determine the relationship between 25(OH)D and blood pressure. Scragg *et al*. [39] randomized 189 elderly subjects who received a single dose (2.5 mg) of cholecalciferol or placebo during the winter. There was no effect on blood pressure. Likewise, our study, conducted in a large sample of individuals with different degrees of obesity, did not show any independent relationship between 25(OH)D concentrations and the odds of having diabetes and/or hypertension.

Vitamin D status has also been inversely related to atherogenic dyslipidemia in some studies [13,40] but not others [31,34,41]. For example, plasma triglyceride concentrations were lower in US adult individuals in the top quartile of 25(OH)D than in those in the reference quartile [40]. A positive association has also been observed between 25(OH)D and plasma HDL-cholesterol concentrations [42]. No RCTs have been conducted to directly analyze the effect of vitamin D supplementation alone on lipid profile. However, a significant decrease in LDL-cholesterol and a non-significant increase in HDL-cholesterol concentrations have recently been observed after an 18-month period of oral vitamin D3 supplementation in Saudi T2DM individuals [43]. Treatment with cholecalciferol associated with energy restriction resulted in a more pronounced decrease in plasma triglyceride concentrations than energy restriction alone [44]. However, long term supplementation with low doses of cholecalciferol (5 to 20 μg) in healthy individuals had no effect on lipid profile, probably because the doses administered were not sufficient to achieve a clinically meaningful effect on lipids [45]. Other RCTs that administered vitamin D supplements and focused on insulin sensitivity/release and blood pressure as the main variables reported no significant effects on lipids [35,36].

Our study demonstrated that there was an association between 25(OH) concentrations and the hypertriglyceridemia component of the MetS, even after adjusting for several confounders. It also showed an association between plasma 25(OH)D concentrations and atherogenic dyslipidemia after adjusting for such potential confounding variables as BMI, which suggests that 25(OH)D status may play a role in lipid profile. This association could be mediated by inflammation, because it disappeared when uCRP was introduced as a covariable in the analysis.

Our results confirm the results of other studies that show that obese individuals with higher BMI have a higher risk of vitamin D deficiency and elevated PTH serum concentrations. When 25(OH)D deficiency and insufficiency were merged, only 38% of individuals with a BMI <30 kg/m^2^ had 25(OH)D insufficiency or deficiency, compared to 88-95% of those with a BMI>35 kg/m^2^. The prevalence of hyperparathyroidism increased from 12% in non-obese individuals to 47.5% in those with a BMI>50 kg/m^2^. Consistent with our results, several other studies have demonstrated an inverse relationship between BMI and 25(OH)D deficiency for both sexes, and for BMIs ranging from normal to severe obesity. In cases of severe obesity, the vitamin D abnormalities were usually more prominent. Prevalence of 25(OH)D deficiency or hyperparathyroidism has been observed to be as high as >50% or 40%, respectively, in individuals with a BMI>50 kg/m^2^. In overweight or obese women, an increase of 1 kg/m^2^ in BMI has been associated with a decrease of 1.21 nmol/L in 25(OH)D levels [5]. Weight loss has been associated with an increase in peripheral 25(OH)D concentrations [24,46]. Therefore, our data confirm previous reports, and show that the presence of obesity is a strong predictor of hypovitaminosis D and hyperparathyroidism.

One reason for the increase in the prevalence of vitamin D deficiency with increasing adiposity may be the higher prevalence of non alcoholic steatohepatitis (NASH) in obesity and insulin resistance states. Vitamin D3 is converted into 25-OH-vitamin D in the liver, and vitamin D deficiency is common in patients with advanced liver disease. In our study we did not include those patients with advanced liver disease in order to discount the effect of this condition on vitamin D concentrations. In fact, only 1.6% of our population had ALAT values that were twice as high as the normal cut-off values (data not shown).

Some of the strengths of our study are the significant amount of clinical data that were collected in a large population of people with different degrees of obesity, and the adjustment for potential confounders such as age, gender, season of blood sampling, current smoking and BMI. However, our study also has its limitations. First, cross-sectional studies are inherently limited in that they cannot establish cause and effect relationships, and our results may not necessarily be valid in non-obese or non-white populations. Second, we acknowledge that major biases with retrospective cohort studies can impact the recall of former exposure to risk variables. Among the biases that can negatively impact the veracity of this type of study are selection bias and misclassification or information bias as a result of the retrospective aspect. Third, in our study we assumed that all individuals with a BMI higher than 35 kg/m^2^ met the abdominal MetS criterion. This could lead to an overestimation of MetS prevalence in individuals between 35 and 40 kg/m^2^. This overestimation is probably minor, as only 0.5% of the PREDIMED individuals [47] with a BMI between 30 and 35 kg/m^2^ had a waist circumference lower than the criterion established for abdominal obesity (data not shown). However, abdominal obesity may have been underestimated specially in individuals between 25 and 30 kg/m^2^. Finally, considering the diversity of results obtained in previous reports on the relationship between 25(OH)D and MetS, and the strong association found between obesity and MetS in this study, we cannot discount that the effect of BMI may have overwhelmed any marginal effect on MetS that could have been attributed to 25(OH)D.

In conclusion, in our study BMI was the variable that was most strongly associated with plasma 25(OH)D and PTH concentrations. Low plasma 25(OH)D and high PTH concentrations were also associated with an increased risk of MetS, but these associations disappeared after adjustment for BMI. On the other hand, our data support a possible contribution of plasma 25(OH)D to the pathogenesis of hypertriglyceridemia, and atherogenic dyslipidemia through inflammation. New prospective studies will be needed to confirm these findings.

## Abbreviations

25(OH)D: 25-Hydroxy-cholecalcipherol; MetS: Metabolic syndrome; PTH: Parathormone; AD: Atherogenic dyslipidemia; BMI: Body mass index; HDL: High density lipoprotein; LDL: Low density lipoprotein; ALAT: Alanine transaminase; GT: Glutamyl transaminase; uCRP: Ultrasensitive C reactive protein; ESR: Erythrocyte sedimentation rate.

## Competing interests

Authors have no conflict of interest to declare.

## Authors’ contributions

AG, MB and JSS conceived the study. AR, DDC, AB, FS and JSS recruited subjects and collected data. AG, MB and JSS performed data analysis and wrote the manuscript. AG, MB and JSS reviewed/edited the final version of the manuscript. All authors approved the final manuscript.
